# Intracellular Trafficking Pathways of *Edwardsiella tarda*: From Clathrin- and Caveolin-Mediated Endocytosis to Endosome and Lysosome

**DOI:** 10.3389/fcimb.2017.00400

**Published:** 2017-09-06

**Authors:** Zhi-hai Sui, Haijiao Xu, Hongda Wang, Shuai Jiang, Heng Chi, Li Sun

**Affiliations:** ^1^Key Laboratory of Experimental Marine Biology, Institute of Oceanology, Chinese Academy of Sciences Qingdao, China; ^2^Laboratory for Marine Biology and Biotechnology, Qingdao National Laboratory for Marine Science and Technology Qingdao, China; ^3^University of Chinese Academy of Sciences Beijing, China; ^4^State Key Laboratory of Electroanalytical Chemistry, Changchun Institute of Applied Chemistry, Chinese Academy of Sciences Changchun, China

**Keywords:** *Edwardsiella tarda*, endocytosis, endosome, lysosome, cytoskeleton

## Abstract

*Edwardsiella tarda* is a Gram-negative bacterium that can infect a broad range of hosts including humans and fish. Accumulating evidences have indicated that *E. tarda* is able to survive and replicate in host phagocytes. However, the pathways involved in the intracellular infection of *E. tarda* are unclear. In this study, we examined the entry and endocytic trafficking of *E. tarda* in the mouse macrophage cell line RAW264.7. We found that *E. tarda* entered RAW264.7 and multiplied intracellularly in a robust manner. Cellular invasion of *E. tarda* was significantly impaired by inhibition of clathrin- and caveolin-mediated endocytic pathways and by inhibition of endosome acidification, but not by inhibition of macropinocytosis. Consistently, RAW264.7-infecting *E. tarda* was co-localized with clathrin, caveolin, and hallmarks of early and late endosomes, and intracellular *E. tarda* was found to exist in acid organelles. In addition, *E. tarda* in RAW264.7 was associated with actin and microtubule, and blocking of the functions of these cytoskeletons by inhibitors significantly decreased *E. tarda* infection. Furthermore, formaldehyde-killed *E. tarda* exhibited routes of cellular uptake and intracellular trafficking similar to that of live *E. tarda*. Together these results provide the first evidence that entry of live *E. tarda* into macrophages is probably a passive, virulence-independent process of phagocytosis effected by clathrin- and caveolin-mediated endocytosis and cytoskeletons, and that the intracellular traffic of *E. tarda* involves endosomes and endolysosomes.

## Introduction

Many serious pathogenic bacteria are capable of invading eukaryotic cells and existing as intracellular parasites (Moulder, 1985; Finlay et al., 1991; Jones and Falkow, 1996; Pieters, 2001). Cellular infection starts with bacterial adhesion to host cells, which results in internalization, either by phagocytosis or by bacteria-induced endocytosis (Finlay and Cossart, 1997). Once entrapped into the vacuoles in host cells, intracellular pathogens can take different pathways for further infection (Clemens et al., 2004). Some bacteria (e.g., *Salmonella typhimurium*) can thrive within the acidic vacuoles that fuse with lysosomes, while others (e.g., *Mycobacterium tuberculosis*) prevent fusion of the pathogen-containing vacuoles with lysosomes, thus maintaining a protected niche inside the host cell; still others (e.g., *Listeria monocytogenes*) can escape from the vacuoles and survive in the cytoplasm (Horwitz, 1988; Garcia-del Portillo and Finlay, 1995). These strategies enable the pathogens to avoid the bactericidal mechanisms of host cells and facilitate the survival and invasion of the pathogens (Gaillard et al., 1987; Clemens, 1996; Rathman et al., 1996; Ray et al., 2009; Seto et al., 2011).

*Edwardsiella tarda*, a Gram-negative bacterium, is a pathogen with a wide host range that includes mammals, fish, birds, and reptiles (Leung et al., 2012). *E. tarda* has been reported to infect humans and cause bacteremia and other medical conditions (Hirai et al., 2015). In aquaculture, *E. tarda* is a severe pathogen and known to affect a large number of farmed fish, resulting in heavy economic losses (Park et al., 2012). *E. tarda* is an intracellular pathogen with the ability to invade and replicate in host phagocytes and non-phagocytes, which is a crucial part of pathogenicity (Janda et al., 1991; Ling et al., 2000; Rao et al., 2001; Okuda et al., 2006; Ishibe et al., 2008; Leung et al., 2012; Wang et al., 2013). Recent studies showed that as a strategy of intracellular survival, *E. tarda* inhibits the apoptosis process of zebrafish cells but induces apoptosis and pyroptosis of mouse macrophages (Zhang et al., 2016; Zhou and Sun, 2016; Qin et al., 2017). In addition, reports have shown that once inside host cells, *E. tarda* could escape from the endocytic vacuoles and replicate in the cytoplasm before releasing from the cells (Strauss et al., 1997). However, the pathways involved in the process of *E. tarda* infection in host cells are unclear.

In this study, we aimed to gain insights into the intracellular infection process of *E. tarda*. For this purpose, we investigated the entry and intracellular transport of *E. tarda* in a mouse macrophage cell line, RAW264.7. Our results indicate a clear preference of *E. tarda* for certain endocytic pathways and an involvement of endosome, lysosome, and cytoskeletons in the infection process.

## Materials and methods

### Reagents and antibodies

The inhibitors used in this study are as follows. Chlorpromazine and sucrose inhibit clathrin-mediated endocytosis; methyl-β-cyclodextrin (MβCD) and nystatin inhibit caveolin-mediated endocytosis; rottlerin and NSC23766 inhibit macropinocytosis; chloroquine and bafilomycin A1 inhibit acidification of endosomes; cytochalasin D and CK-636 inhibit actin polymerization; nocodazole and vinblastine depolymerize microtubles. All inhibitors were purchased from Selleck (USA) and Sigma-Aldrich (USA). All inhibitors, except sucrose, were dissolved in dimethyl sulfoxide (DMSO) (Sigma, USA) according to the manufacturer's instructions. Tubule-Tracker red kit and Lyso-Tracker red kit was purchased from Beyotime Biotechnology (Beijing, China). Fluorescein isothiocyanate (FITC), 4′-6-diamidino-2-phenylindole (DAPI), formaldehyde and paraformaldehyde (PFA) was purchased from Solarbio (Beijing, China). Latex beads (1 μm) were purchased from Polysciences (USA). Mouse monoclonal antibody against clathrin heavy chain and caveolin-1, rabbit polyclonal antibodies against rab5, lamp1, and cathepsin D, phalloidin-iFluor 594 Reagent and Alexa Fluor 594-conjugated secondary antibodies were purchased from Abcam (UK) and ABclonal (USA). Rat polyclonal antibodies against *E. tarda* have been reported previously (Zhou and Sun, 2016).

### Cell line

RAW264.7, a murine monocyte-macrophage cell line, was purchased from American Tissue Culture Collection (ATCC, USA). The cells were cultured in Dulbecco's minimal Eagle's medium (DMEM) (Gibco, USA) containing 10% fetal bovine serum (FBS) (Gibco, USA) at 37°C in 5% CO_2_.

### Bacteria

*E. tarda* TX1 (Zhang et al., 2008) was cultured in Luria–Bertani broth (LB) medium at 28°C. TX1 was transformed with the plasmid pGFP_UV_ (purchased from Clonetech, USA), and the transformant was named TX1G, which exhibits ampicillin resistance (marker of pGFP_UV_) and green fluorescence under UV light. To examine the stability of TX1G, the bacteria were sub-cultured continuously in LB medium without ampicillin for 7 times, and the bacteria were examined for pGFP_UV_ presence and observed with a fluorescence microscope. The serum survival and 50% lethal dose (LD_50_) of TX1G were determined as reported previously (Yan et al., 2012).

### Intracellular replication of *E. tarda*

*E. tarda* TX1G was grown in LB medium at 28°C to an OD_600_ of 0.7. The bacteria were collected by centrifugation, washed with PBS, and resuspended in PBS. The bacteria were added to 100% confluent RAW264.7 cells in a 24-well plate at a multiplicity of infection (MOI) of 10:1, and the plate was centrifuged at 800 g for 10 min, followed by incubation at 28°C for 2 h. Extracellular *E. tarda* was killed by adding gentamicin (100 μg/ml) to the plate, followed by incubation at 28°C for 1 h. The cells were washed three times with PBS and cultured in DMEM containing 10 μg/ml gentamicin for 0, 2, 4, 6, and 8 h. At each time point, 500 μl 1% Triton X-100 was added to the plate to lyse the cells, and the lysate was diluted and plated onto LB agar plates supplemented with 30 μg/ml tetracycline (one of the antibiotic resistance markers of TX1G). The plates were incubated at 28°C for 48 h, and the colony-forming units (CFU) were counted. The colonies were verified to be TX1G by PCR. The experiment was performed in triplicate.

### Effects of inhibitors on *E. tarda* infection

RAW264.7 was cultured to 100% confluence in 24-well plates. The cells were incubated with sucrose (300 mM), chlorpromazine (20 μM), MβCD (1 mM), nystatin (100 μM), rottlerin (40 μM), NSC23766 (100 μM), chloroqiune (40 μM), bafilomycin A1 (1.6 μM), cytochalasin D (2 μM), CK-636 (50 μM), nocodazole (2 μM), or vinblastine (100 μM) for 1 h. The cells were then infected with *E. tarda* TX1G at a MOI of 10 at 28°C for 2 h. The cells were washed three times with PBS and treated with 100 μg/ml gentamicin for 1 h. After treatment, the cells were washed and lysed as above. Viable bacteria in the lysate were determined by plate count as above.

### Transfection of Raw264.7 with siRNAs

All siRNAs were obtained from Ribobio (Guangzhou, China). RAW264.7 cells were cultured to about 30–40% confluence in 24-well plates. Transfection was performed with Lipofectamine® 2000 Reagent Protocol (invitrogen, USA) according to the instructions of the manufacturer. Briefly, the cells were transfected with 50 nM siRNAs of clathrin heavy chain or caveolin-1 (Zhu et al., 2011) or a scrambled siRNA (a negative control) in Lipofectamine 2000 and Opti-MEM (Invitrogen, USA) for 6 h. The medium was then replaced with DMEM containing 10% FBS, and the plate was incubated at 37°C with 5% CO_2_ for an additional 48 h. The expression of clathrin and caveolin-1 was determined by quantitative real time reverse transcription-PCR (qRT-PCR). The primers for PCR clathrin were Clathrin-RT-F and Clathrin-RT-R, and the primers for PCR caveolin were Caveolin-RT-F and Caveolin-RT-R (Table S1). *E. tarda* infection of the cells was performed as above.

### Fluorescence activated cell sorting (FACS)

RAW264.7 cells were treated with chlorpromazine or nystatin as above. The cells were then infected with live *E. tarda* TX1G as above or incubated with formaldehyde-killed *E. tarda* TX1G (MOI of 10:1) at 28°C for 2 h. The cells were washed with PBS for three times, and extracellular fluorescence was quenched by adding 1 ml 0.125% trypan blue in PBS, followed by incubation at 22°C for 30 min. The cells were washed as above and suspended in 1 ml PBS. The cells were subjected to flow cytometry analysis with a Partec CyFlow Counter (Partec GmbH, Munster, Germany), and the data were analyzed with FlowJo 7.6.1 software (TreeStar, San Carlos, CA, USA).

### Immunofluorescence microscopy

RAW264.7 was incubated with *E. tarda* TX1G or formaldehyde-fixed *E. tarda* TX1G in 35 mm confocal dishes for different time at 28°C. The cells were fixed with 4% PFA for 20 min and permeabilized with 0.1 % Triton-X-100 for 10 min, followed by incubation with 5% BSA for 4 h. The cells were stained with the rabbit or mouse primary antibodies (1:500) for overnight at 4°C. After washing three times with PBS, the cells were incubated with secondary antibodies (Alexa Fluor 594-conjugated goat anti-mouse or rabbit IgG) (1:500) for 90 min at room temperature. The cells were washed as above and stained with DAPI for 10 min at room temperature. The cells were washed as above and observed with a confocal microscope (Zeiss LSM 710). At least 100 individual bacteria were scored for co-localization in at least five random fields for each experiment. Percentages of co-localization are the average of at least three experiments.

### Immunofluorescence observation of intracellular and extracellular *E. tarda*

RAW264.7 was infected with *E. tarda* TX1G, washed with PBS, and fixed with PFA as above. For immunofluorescence staining of extracellular bacteria, rat anti-*E. tarda* antibody (1:1000) was added to the cells, and the cells were incubated at 22°C for 2 h. The cells were washed as above, and goat anti-rat IgG conjugated to rhodamine red (1:1000) was added to the cells. The cells were incubated at 22°C for 2 h and washed as above. The cells were observed with a confocal microscope (Zeiss LSM 710), and intracellular and extracellular bacteria were distinguished by different colors (Drevets and Campbell, 1991; Campbell et al., 2001).

### Lyso-tracker red labeling

RAW264.7 were infected with *E. tarda* TX1G for 1.5 h as described above, and the cells were washed with PBS and incubated with 50 nM Lyso-Tracker red in DMEM containing 10 % FBS for 30 min at 28°C. The cells were washed, fixed, and stained with DAPI as above. The cells were washed with PBS and viewed with a confocal microscope as above.

### Labeling of actin and microtubules

RAW264.7 cells were incubated with *E. tarda* TX1G or latex beads for 1.5 h as described above. The cells were fixed with 4% PFA for 20 min and permeabilized with 0.1% Triton-X-100 for 10 min, followed by incubation with 5% BSA for 4 h. The cells were stained with Phalloidin-iFluor 594 Reagent (1:1,000) or Tubule-Tracker red (1:500) for 30 min at room temperature. The cells were washed and stained with DAPI as above. The cells were washed with PBS and viewed with a confocal microscope as above.

### Statistical analysis

All experiments were performed at least three times, and statistical analyses were carried out with SPSS 17.0 software (SPSS Inc., Chicago, IL, USA). Data were analyzed with analysis of variance (ANOVA), and statistical significance was defined as *P* < 0.05.

## Results

### Stability of GFP-expressing *E. tarda*

To facilitate the study, the GFP-expressing plasmid pGFP_UV_ was introduced into *E. tarda* TX1, resulting in strain TX1G. pGFP_UV_ was maintained stably in TX1G after 7 subcultures without selective pressure, and the cells exhibited strong fluorescence easily detectable by microscopy (Figure S1). Compared to *E. tarda* TX1, TX1G exhibited similar LD_50_ (9.3 × 10^3^ CFU/g) and serum survival rate, suggesting that the introduced pGFP_UV_ had no apparent effect on the virulence property of the bacteria. As such, TX1G was used for all experimental analyses in this study.

### Intracellular replication of *E. tarda* in RAW264.7

**Figure 1:**
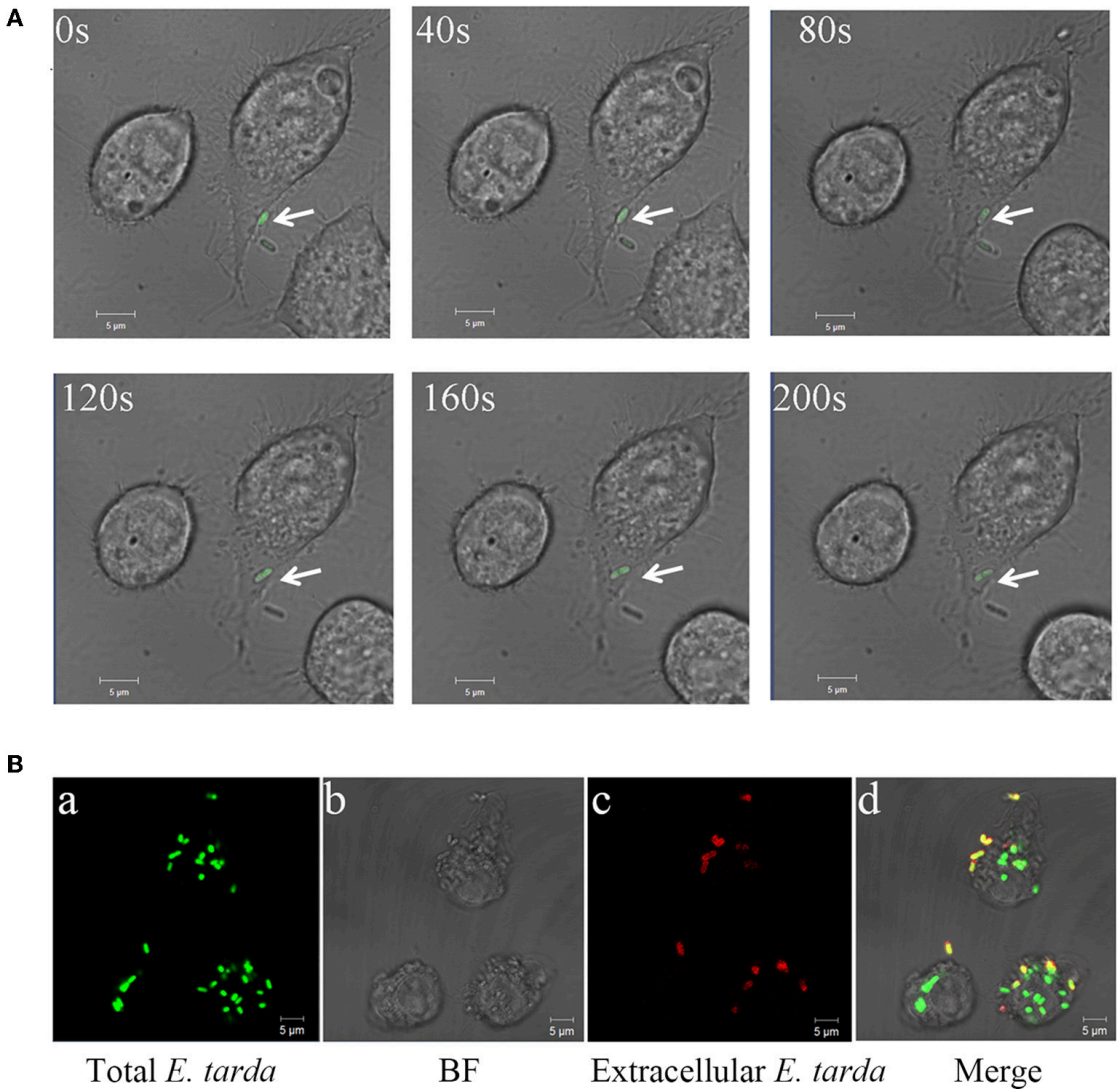
Entry and replication of *Edwardsiella tarda* in RAW 264.7. **(A)** RAW264.7 was incubated with *E. tarda* TX1G, and infection of the bacteria into RAW264.7 was observed continuously for 200 s with a confocal microscope. Arrow indicates bacteria. **(B)** RAW264.7 was infected with TX1G for 2h **(a)**; the cells were then fixed, and extracellular TX1G was detected with rhodamine-labeled antibody **(c)**. **d** merge of **a–c**.

To assess the ability of *E. tarda* TX1G to replicate in host cells, the bacteria were incubated with RAW264.7, and intracellular bacterial number was determined at different times. The results showed that the intracellular number of TX1G increased steadily with time from 2 to 8 h (Figure S2). Consistently, microscopic analysis observed entry of the bacteria into RAW264.7 and, after 2 h of incubation, an apparent accumulation of bacterial load inside RAW264.7 (Figure 1). As shown in Figure 1Bd, intracellular bacteria (green) were easily distinguishable from extracellular bacteria (yellow) by color.

### Involvement of the endocytic pathway in *E. tarda* infection

**Figure 2:**
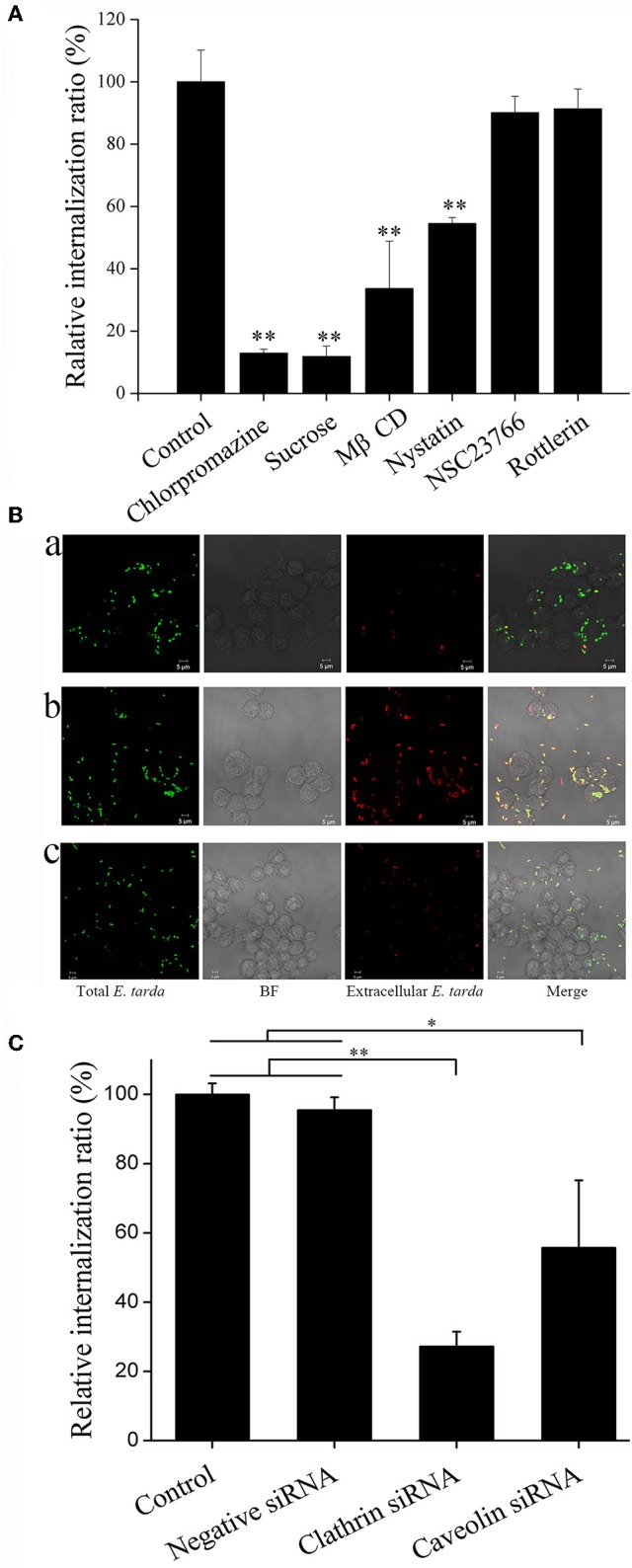
Effect of endocytic pathway inhibitors and siRNAs on *Edwardsiella tarda* infection. **(A)** RAW264.7 was infected with *E. tarda* TX1G in the presence or absence (control) of various endocytic pathway inhibitors, and cellular internalization of the bacteria was determined by plate count. **(B)** RAW264.7 was infected with TX1G in the presence of chlorpromazine **(b)** and nystatin **(c)**; the control cells were infected with TX1G without any inhibitor **(a)**. The cells were fixed, and extracellular bacteria were detected with rhodamine-labeled antibody (red color). The cells were observed with a confocal microscope. Bar: 5 μm. **(C)** RAW264.7 was transfected with siRNAs of clathrin/caveolin-1 or negative control siRNAs before *E. tarda* infection, and cellular internalization of the bacteria was determined by plate count. **(A)** and **(C)**, data are the means of three assays and presented as means ± SEM. ^*^P < 0.05, ^**^P < 0.01.

To examine the pathways involved in *E. tarda* entry into host cells, TX1G infection of RAW264.7 was conducted in the presence of inhibitors against clathrin (chlorpromazine and sucrose), caveolin (MβCD and nystatin), and macropinocytosis (NSC23766 and rottlerin). Subsequent analysis showed that in the presence of chlorpromazine, sucrose, MβCD, and nystatin, the relative infection ratio of TX1G decreased to 12.9, 11.8, 33.6, and 54.5, respectively, whereas the presence of NSC23766 and rottlerin had no significant effect on *E. tarda* infection (Figure 2A). Consistently, microscopic examination indicated that the presence of nystatin and, in particular, chlorpromazine, severely decreased the number of internalized bacteria in RAW264.7 (Figure 2B). To verify the importance of clathrin- and caveolin-mediated endocytosis for *E. tarda* infection, the expression of clathrin and caveolin in RAW264.7 was interfered by siRNAs, which, as shown by qRT-PCR, significantly decreased the expression of clathrin and caveolin to 24.3 ± 8.3 and 38.6 ± 6.7% of that in the control cells, respectively. Subsequent infection analysis showed that in RAW264.7 with clathrin and caveolin knock-down, the uptake of *E. tarda* was reduced to 27.2 ± 4.2 and 55.7 ± 1.9% of that in the control cells, respectively (Figure 2C). These results indicated that clathrin- and caveolin-mediated pathways were vital to *E. tarda* infection. To examine whether these pathways were also involved in the uptake of dead bacteria, FACS analysis was performed, which showed that the presence of chlorpromazine and nystatin significantly reduced the cellular uptake of formaldehyde-killed *E. tarda* to 67.1 ± 5.1 and 73.8 ± 3.0% of that of the control, respectively, which were roughly similar to the reduction rates (70.2 ± 4.5 and 75.5 ± 4.5%) in the uptake of live *E. tarda* (Figure S3).

### Co-localization of clathrin and caveolin with invading *E. tarda*

**Figure 3:**
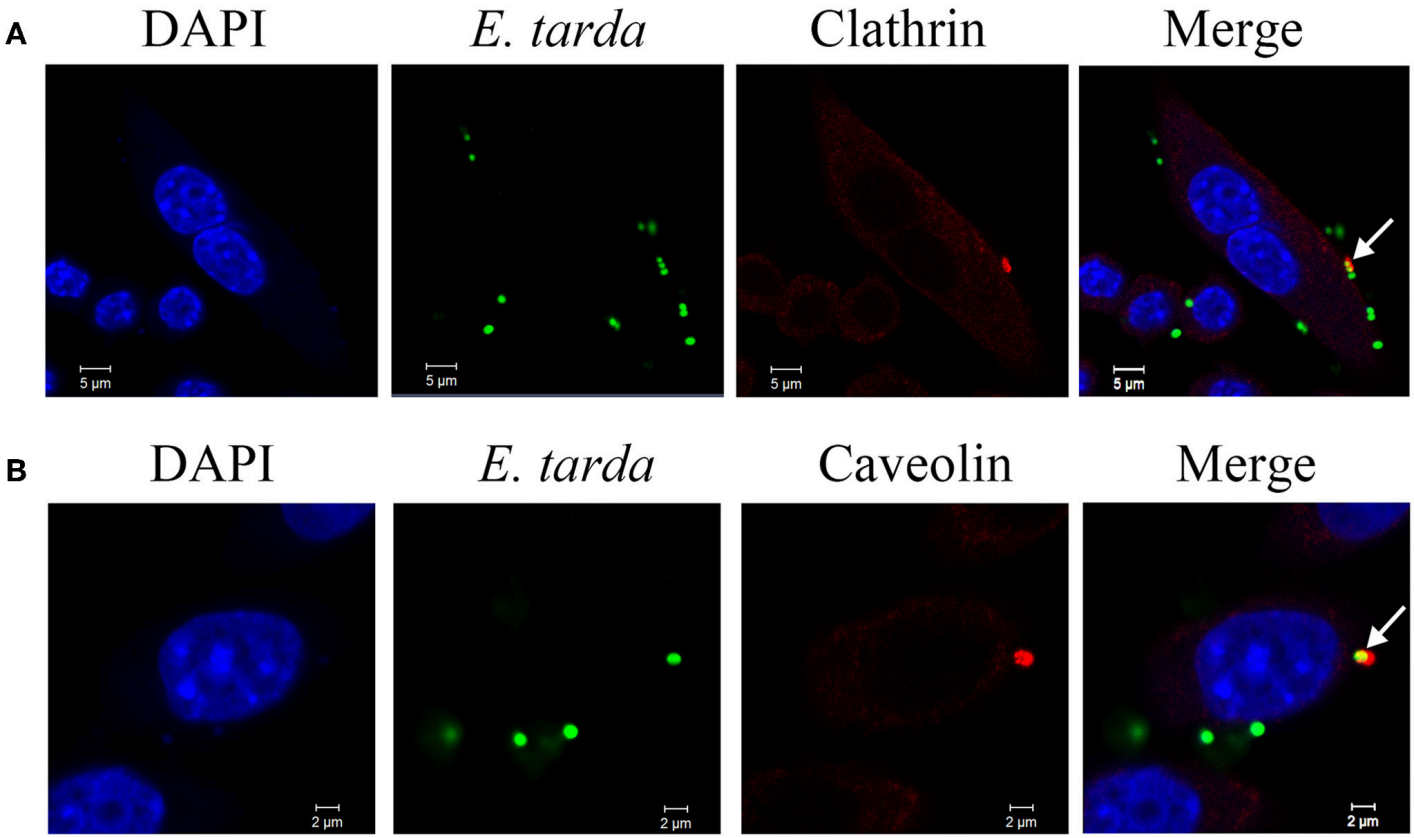
Co-localization of *Edwardsiella tarda* with clathrin **(A)** and caveolin **(B)** during bacterial infection of RAW264.7. RAW264.7 cells infected with *E. tarda* TX1G for 30 min were treated with Alexa Fluor594-labeled antibody against clathrin or caveolin. The cells were stained with DAPI and observed with a confocal microscope. White narrows indicate co-localization of bacteria and clathrin/caveolin.

Since the above results indicated a requirement of clathrin-and caveolin-mediated pathways for *E. tarda* invasion, we examined whether *E. tarda* was associated with clathrin and caveolin. For this purpose, immunofluorescence microscopy was performed to examine the locations of clathrin/caveolin and *E. tarda* during bacterial infection of RAW264.7. The results showed that both clathrin and caveolin were co-localized with TX1G (Figure 3). The co-localization rates of bacteria with clathrin and caveolin were 23.6 ± 6.4 and 20.4 ± 7.4%, respectively. In addition, clathrin and caveolin distribution in uninfected control cells was also observed (Figure S4).

### Involvement of endosome/lysosome in *E. tarda* infection

**Figure 4:**
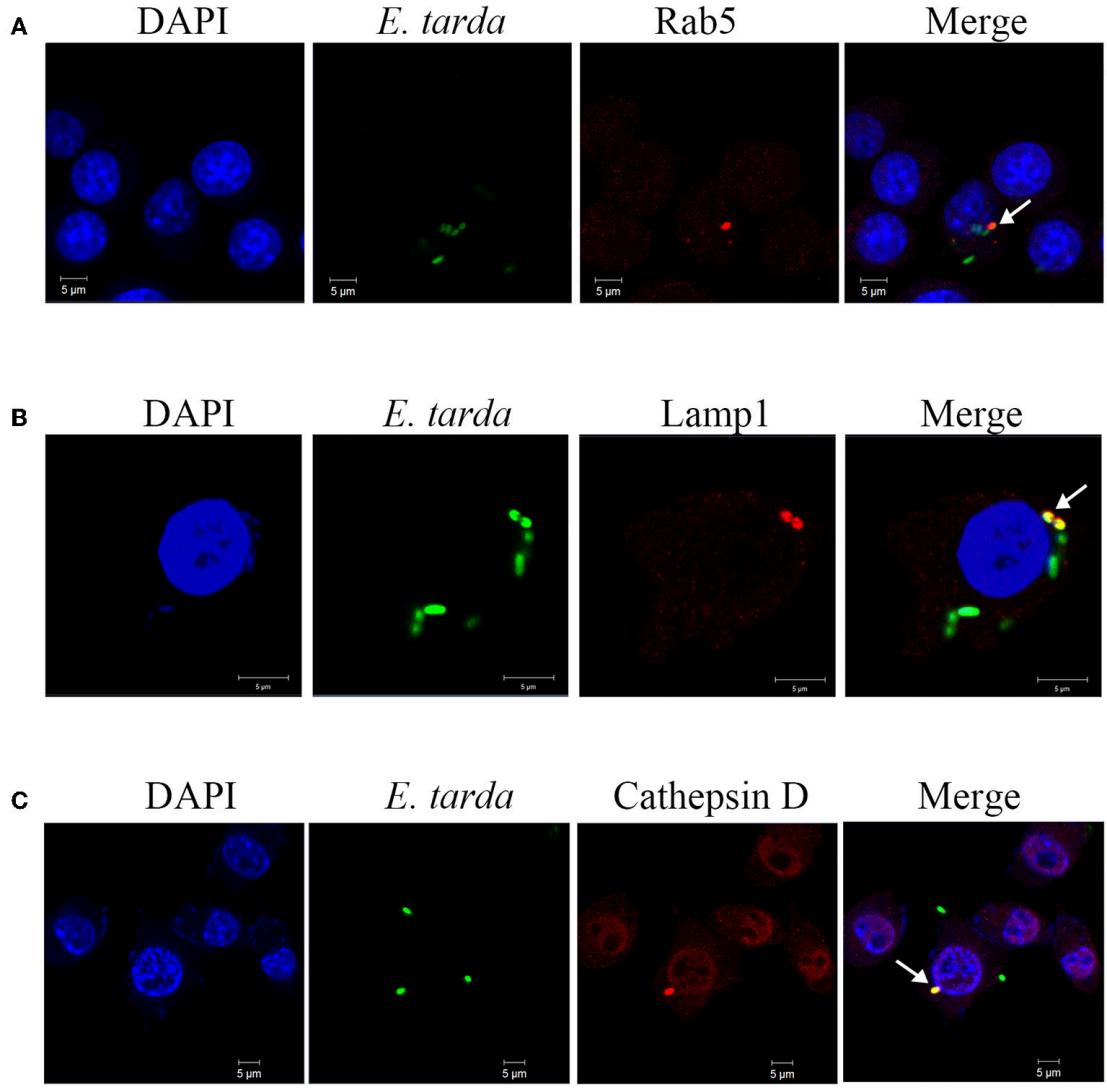
Co-localization of *Edwardsiella tarda* with early and late endosome markers. RAW264.7 cells infected with *E. tarda* TX1G for 1 h **(A)** or 1.5 h **(B,C)** were treated with Alexa Fluor594-labeled antibody detecting Rab5 **(A)**, Lamp1 **(B)**, or cathepsin D **(C)**. The cells were stained with DAPI and observed with a confocal microscope. White arrows indicate co-localization of bacteria and the respective marker.

**Figure 5:**
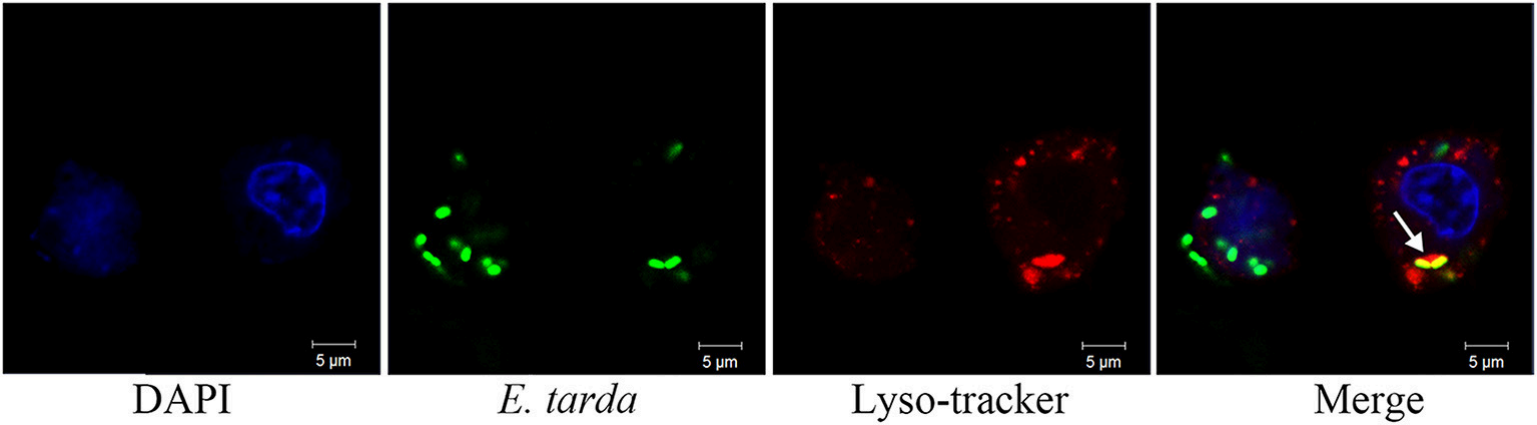
Co-localization of *Edwardsiella tarda* with lysosomes in RAW264.7. RAW264.7 cells infected with *E. tarda* TX1G for 1.5 h were treated with Lyso-tracker red. The cells were stained with DAPI and observed with a confocalmicroscope. White arrow indicates co-localization of bacteria and lysosome.

To examine the potential role of endosomes in *E. tarda* infection, RAW264.7 was infected with TX1G in the presence of chloroquine and bafilomycin A1, inhibitors of endosome acidification. Subsequent analysis of bacterial infection showed that the relative infection ratio of TX1G decreased to 47 ± 8 and 46 ± 9% of that of the control by chloroquine and bafilomycin A1, respectively. Microscopy showed that in TX1G-infected RAW264.7, the bacteria were co-localized with Rab5, a marker of early endosomes, and Lamp1 and cathepsin D, markers of late endosomes/lysosomes (Figure 4). The percentages of bacterial co-localization with Rab5, LAMP1 and cathepsin D were 29.6 ± 6.4%, 28.7 ± 8.5%, 36.7 ± 7.6%, respectively. Similar observation was made with formaldehyde-killed *E. tarda* (Figure S5). To further examine the association of lysosome with TX1G infection, TX1G-infected RAW264.7 was treated with Lyso-tracker red, an indicator of acid environment, and subsequent microscopy showed that co-localization of the red tracker with TX1G was detected (Figure 5).

### Involvement of cytoskeletons in *E. tarda* infection

**Figure 6:**
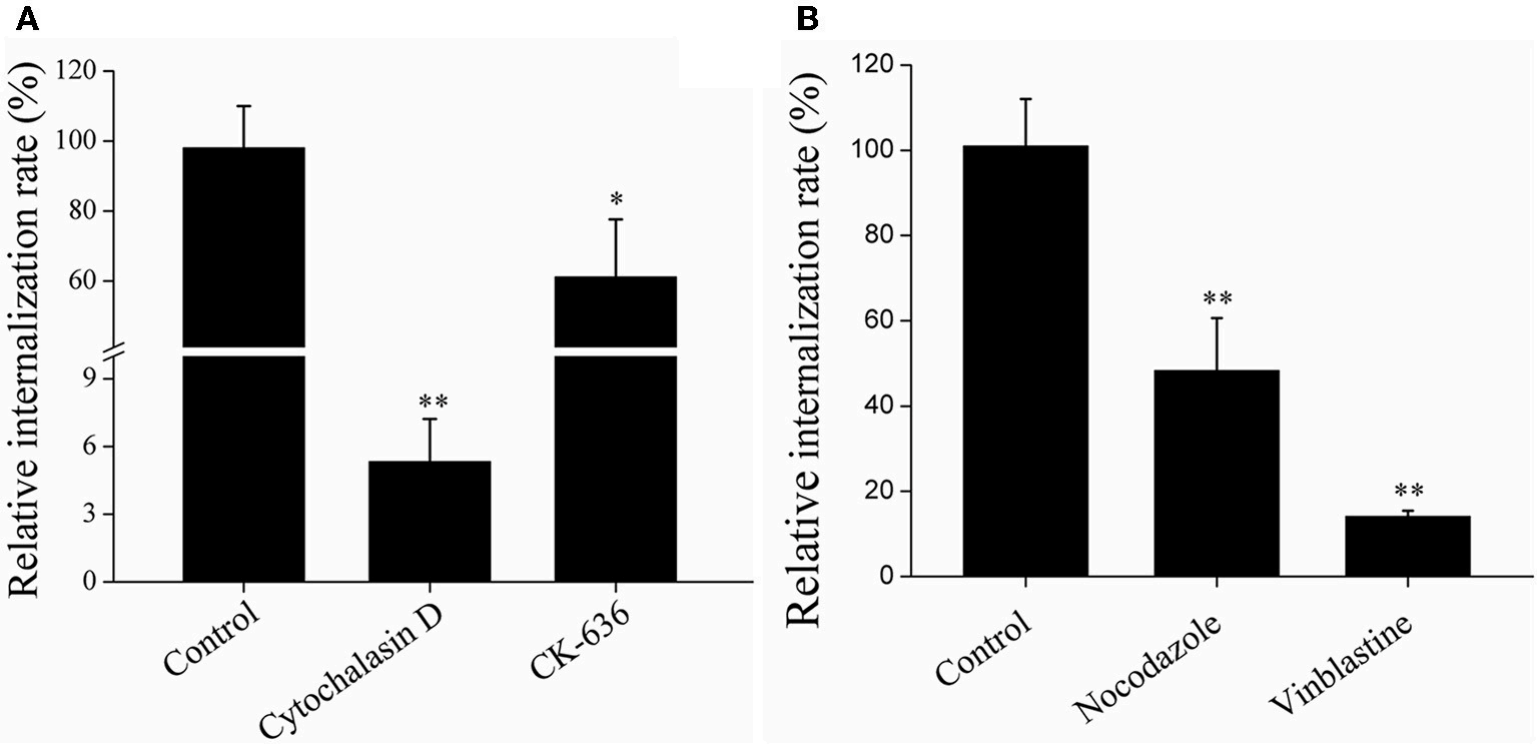
Effects of actin **(A)** and microtubule **(B)** inhibition on *Edwardsiella tarda* infection. RAW264.7 was infected with *E. tarda* TX1G in the presence or absence (control) of the inhibitors against actin **(A)** or microtubule **(B)**, and cellular internalization of the bacteria was determined by plate count. Data are the means of three independent experiments and presented as means ± SEM. ^*^P < 0.05, ^**^P < 0.01.

**Figure 7:**
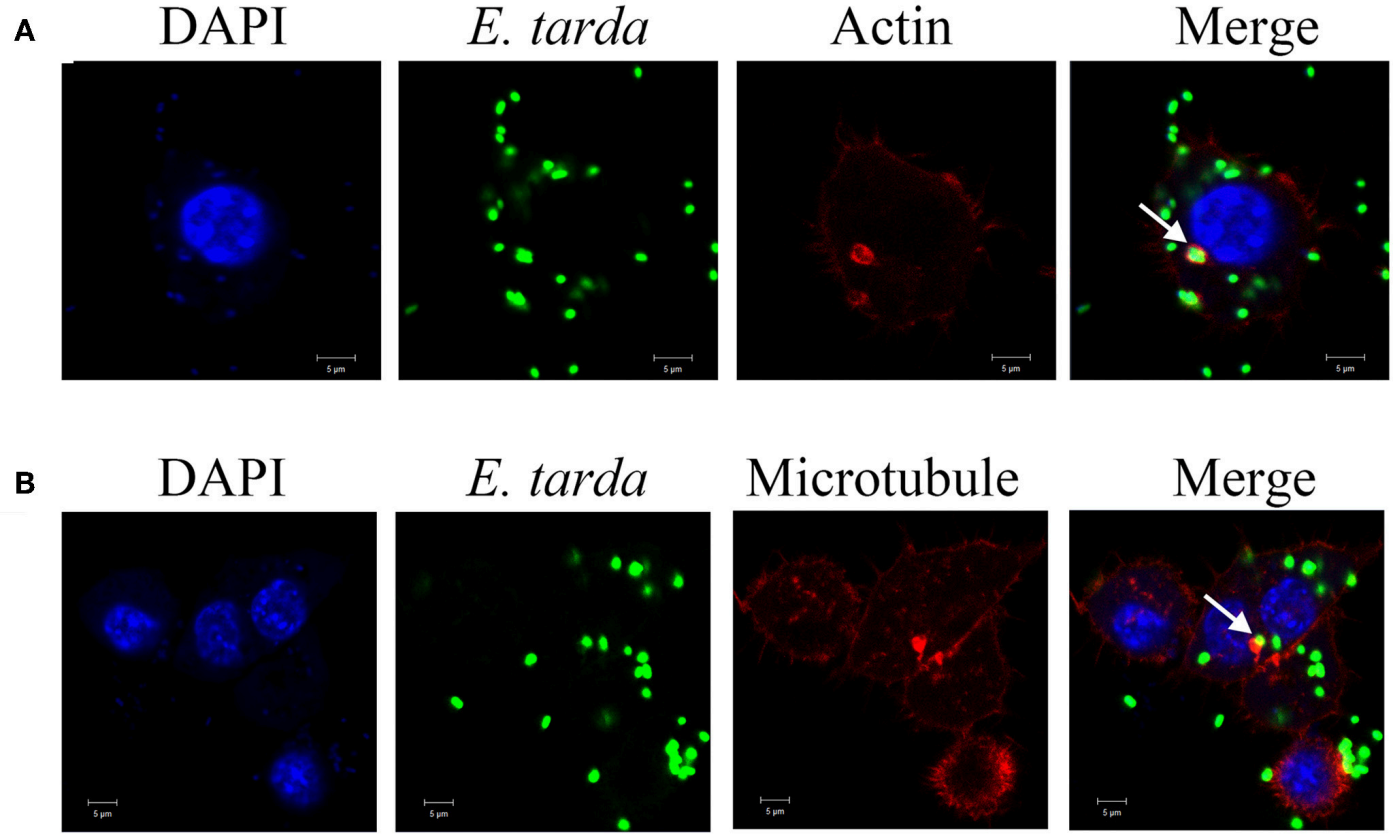
Co-localization of actin **(A)** and microtubule **(B)** with *Edwardsiella tarda* in RAW264.7. RAW264.7 cells infected with *E. tarda* TX1G were treated with Alexa Fluor594-labeled phalloidin **(A)** or Tubulin-Tracker Red **(B)**. The cells were then observed with a confocal microscope. White arrow indicates co-localization of bacteria and actin/microtubule. Bar: 5 μm.

To examine whether cytoskeletons were required for *E. tarda* infection, RAW264.7 was infected with TX1G in the presence of actin inhibitors (cytochalasin D and CK-636) and microtubule inhibitors (nacodazole and vinblastine). Subsequent infection analysis showed that the presence of these inhibitors significantly decreased the infection ratio of TX1G to 5.3, 61.1, 48.3, and 14.1%, respectively, of that of the control (Figure 6). Consistently, immunofluorescence microscopy indicated that TX1G was co-localized with phalloidin-marked actin and Tubulin-Tracker Red (Figure 7). When TX1G was replaced with latex beads, co-localization of the beads with actin and microtubule was also observed (data not shown).

## Discussion

Survival in host phagocytes is an important virulence property for intracellular bacterial pathogens (Finlay and Falkow, 1997). For *E. tarda*, several studies have demonstrated that it is able to replicate in various types of host cells including fish and mouse macrophages (Okuda et al., 2006; Zhang et al., 2016; Qin et al., 2017). Likewise, in this study we found that *E. tarda* TX1G replicated steadily in RAW264.7 in a time-dependent manner, indicating an immune evasion capacity of TX1G against the bactericidal activities of macrophages. This result is consistent with our previous observation that following infection into host cells, *E. tarda* induces a cellular process that inhibits the initiation of apoptosis, thus enabling efficient bacterial dissemination in the host (Zhou and Sun, 2016).

Macrophages play an essential role in the immune response against pathogens (Johnston, 1988; Fujiwara and Kobayashi, 2005). Macrophages engulf microorganisms as well as other foreign particles by several mechanisms, which include phagocytosis, macropinocytosis, clathrin- and caveolin-mediated endocytosis, and clathrin- and caveolin-independent endocytosis (Conner and Schmid, 2003). Pharmacological inhibitors are often used to investigate which endocytic mechanism is responsible for cellular uptake of particles (Iversen et al., 2011). In our study, we found that of the six inhibitors examined, the four inhibitors against clathrin and caveolin significantly decreased the infection of *E. tarda*; of these four inhibitors, chlorpromazine and sucrose targeting clathrin induced much higher levels of inhibition than MβCD and nystatin targeting caveolin, suggesting that endocytosis mediated by clathrin and caveolin, especially the former, is vital to *E. tarda* infection. This conclusion was supported by microscopic observation, which showed co-localizations of *E. tarda* with clathrin and caveolin. In addition to live *E. tarda*, clathrin and caveolin pathways were also involved in the uptake of dead *E. tarda*, suggesting that these pathways were not specifically invoked by *E. tarda* during infection, but rather operated as common pathways during the phagocytosis process. Previous studies have shown that macropinocytosis is utilized by some pathogens, such as *Salmonella*, in their infection of target cells (Kerr et al., 2010). In our study, we found that unlike the inhibitors of clathrin and caveolin, the two inhibitors of macropinocytosis failed to exert any significant effect on *E. tarda* infection, suggesting that macropinocytosis is probably not involved in the intracellular infection of *E. tarda*.

After entry into host cells, bacterial pathogens can undergo different intracellular lifestyles, such as localizing in the cytosol or being sequestered in vesicular structures (Finlay and Cossart, 1997; Clemens et al., 2004; Bhavsar et al., 2007). For example, *Legionella pneumophila* entered vacuoles and inhibited phagosome acidification and maturation (Horwitz and Maxfield, 1984). In our study, we found that *E. tarda* was co-localized with the markers of early endosomes and late endosomes/lysosomes, and that inhibition of endosome acidification significantly decreased the infection of *E. tarda*. Consistently, lyso-tracker red, an indicator of acid environment, was co-localized with *E. tarda*. These results suggested that following internalization into RAW264.7, *E. tarda* was transported from early endosomes to endolysosomes, which is in agreement with the previous reports that showed *E. tarda* existence in the vacuoles of human epithelial cells and mouse macrophages (Fernández et al., 2014; Qin et al., 2017). Since dead *E. tarda* was also co-localized with endosome markers, this intracellular trafficking process is very likely not involving any invasion mechanism of *E. tarda*. However, given the environmental stress within the endolysosomes/phagolysosomes caused by hydrolytic enzymes and low pH (Baltierra-Uribe et al., 2014), the survival of live *E. tarda* in these vacuoles implies possession of certain virulence mechanisms by *E. tarda* against the harsh conditions in the cellular compartments of host phagocytes.

Cytoskeletons provide the necessary force for the uptake of exogenous particles into membrane-bound vacuoles (Finlay and Cossart, 1997). During the process of infection, many pathogenic bacteria are model the structure of cytoskeletons to facilitate host cell infection, and cytoskeleton-dependent internalization mechanisms have been reported for several invasive organisms (Cossart and Kocks, 1994; Hu and Kopecko, 1999; Gruenheid and Finlay, 2003; Yoshida and Sasakawa, 2003; Veiga and Cossart, 2006). For example, *Shigella* is known to secret the VirA effector protein to destabilize microtubule to promote infection (Finlay and Cossart, 1997), and *Salmonella* invades host cells via a mechanism involving actin reorganization (Lim et al., 2014). In our study, we found that destruction of actin and microtubule by inhibitors significantly decreased *E. tarda* infection in RAW264.7, and, consistently, *E. tarda* was co-localized with actin and microtubule, which is in line with the previous observation that *E. tarda* was associated with actin in carp cells (Ling et al., 2000). These results indicated that engulfment of *E. tarda* depends on the normal functioning of cytoskeletons.

In conclusion, we demonstrated for the first time that *E. tarda* enters, most likely in a passive manner, macrophages via the general phagocytosis pathways mediated by clathrin and caveolin, but not by macropinocytosis. Intracellular *E. tarda* is maintained in vacuoles, and transported from early endosomes to endolysosomes. Also, cellular infection of *E. tarda* requires the participation of actin and microtubule of the host cells. These results provide new insights into the intracellular invasion mechanism of *E. tarda*.

## Author contribution

LS and ZS conceived and designed the experiments; ZS, SJ, and HC performed the experiments; HX and HW provided technical support for the experiments. ZS, SJ, and HC analyzed the data; ZS and LS wrote the manuscript. All authors read and approved the final manuscript.

### Conflict of interest statement

The authors declare that the research was conducted in the absence of any commercial or financial relationships that could be construed as a potential conflict of interest.
