# Supplementary material for: Intracellular Trafficking Pathways of Edwardsiella tarda: From Clathrin- and Caveolin-Mediated Endocytosis to Endosome and Lysosome
Source: Front Cell Infect Microbiol. 2017 Sep 6;7:400. doi: 10.3389/fcimb.2017.00400 (PMC5592743; doi:10.3389/fcimb.2017.00400)
Supplement: Supplementary file 1 [file DataSheet1.docx]

**Intracellular trafficking pathways of *Edwardsiella tarda*: from clathrin- and caveolin- mediated endocytosis to endosome and lysosome**

Zhi-hai Sui^1,2,4^, Haijiao Xu^3^, Hongda Wang^3^, Shuai Jiang^1,2^, Heng Chi^1,2^, Li Sun^1,2^*

*^1^ Key Laboratory of Experimental Marine Biology, Institute of Oceanology, Chinese Academy of Sciences, Qingdao, China*

*^2^* *Laboratory for Marine Biology and Biotechnology, Qingdao National Laboratory for Marine Science and Technology, Qingdao, China*

***^3^*** *State Key Laboratory of Electroanalytical Chemistry, Changchun Institute of Applied Chemistry, Chinese Academy of Sciences, Changchun, China.*

*^4^ University of Chinese Academy of Sciences, Beijing, China*

*To whom correspondence should be addressed

Mailing address: Li Sun

Institute of Oceanology

Chinese Academy of Sciences

7 Nanhai Road

Qingdao 266071, China

Phone: 86-532-82898829

Fax: 86-532-82898829

Email:[lsun@qdio.ac.cn](mailto:lsun@qdio.ac.cn)

Running title: Intracellualr infection pathways of *Edwardsiella tarda*

**Supplemental data**

**Figure S1.** Microscopic examination of *Edwardsiella tarda* TX1G. TX1G cells after 7 subcultures were stained with DAPI and examined with a fluorescence microscope.


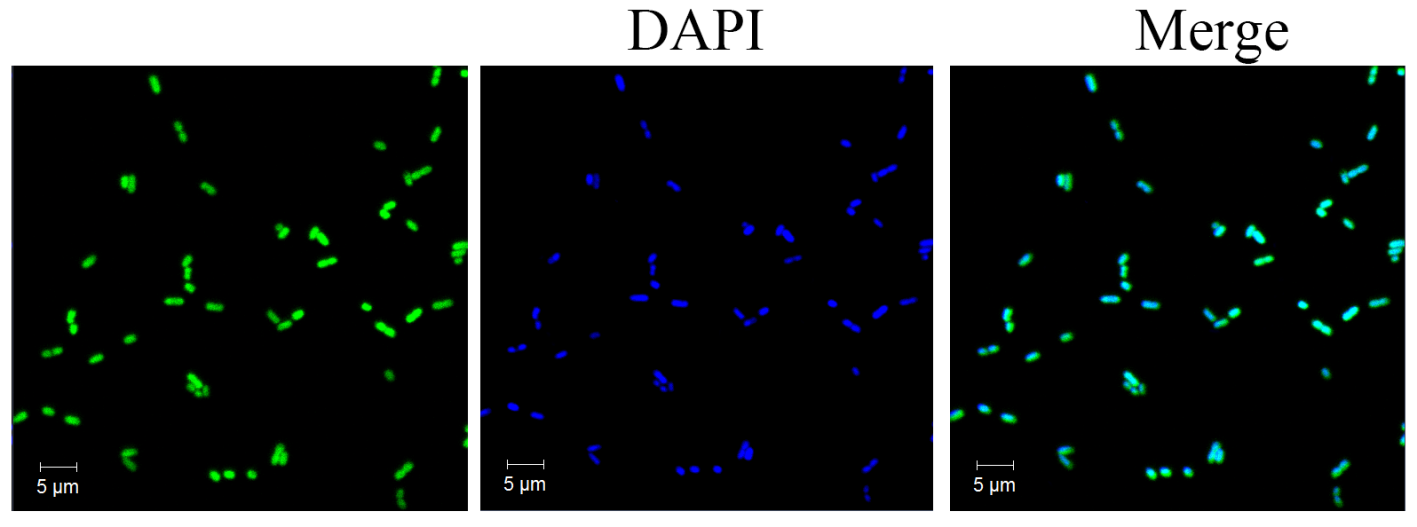


**Figure S2**. Intracellular replication of *Edwardsiella tarda* in RAW 264.7 as determined by plate count. RAW264.7 was infected with *E. tarda* TX1G for 2 h, and the extracellular bacteria were killed. The cells were then incubated for 0 h, 2 h, 4 h, 6 h and 8 h, and the number of intracellular bacteria at each time point was determined by plate count. Data are the means of three independent experiments and presented as means ± SEM.

**

**

**Figure S3.** Effect of endocytic pathway inhibitors on the uptake of live and dead *Edwardsiella tarda*. RAW264.7 cells were incubated with live (A) or dead (B) *E. tarda* TX1G in the presence or absence (control) of chlorpromazine and nystatin. Bacterial uptake into the cells was then determined by flow cytometry. Data are the means of three experiments and presented as means ± SEM.^**^*P* < 0.01.


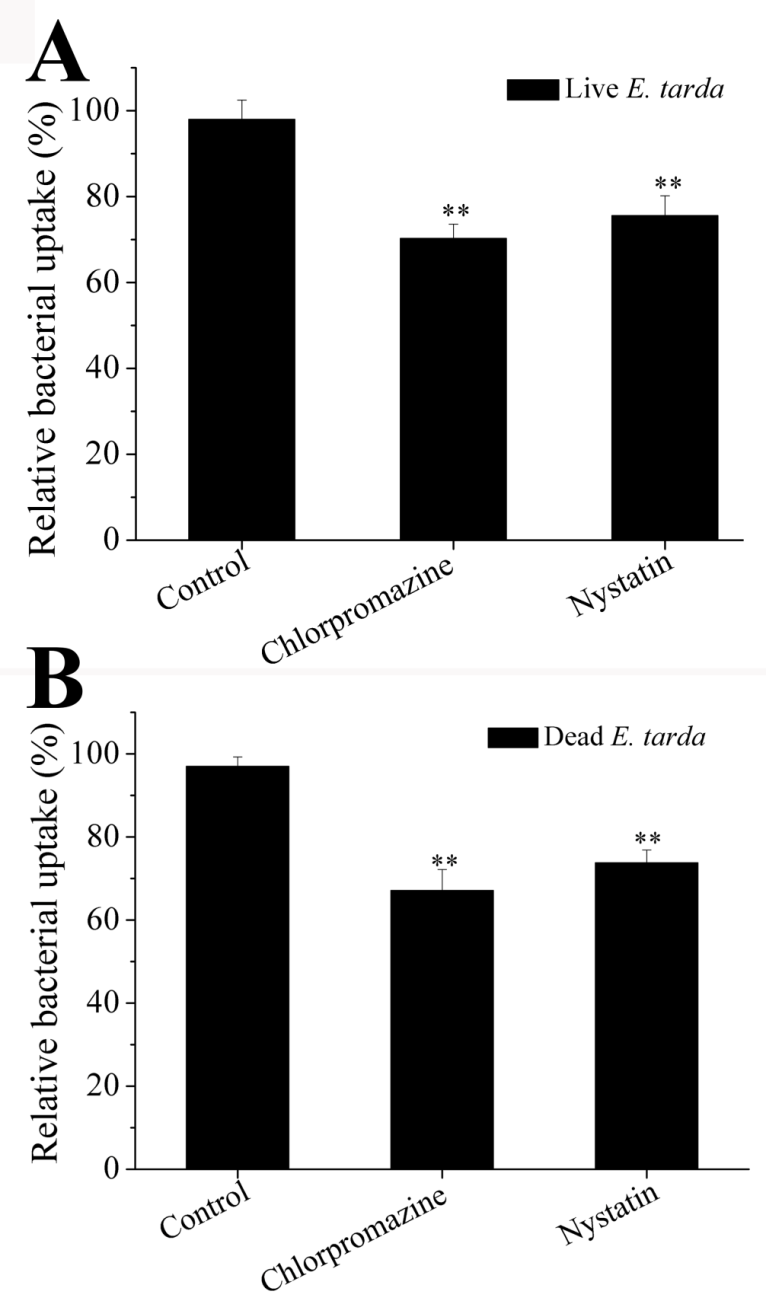


**Figure S4.** Immunofluorescence detection of clathrin (A) and caveolin (B) in RAW264.7. RAW264.7 cells were fixed with 4% PFA and permeabilized with 0.1% Triton-X-100. Clathrin (A) and caveolin (B) were detected with Alexa Fluor594-labeled antibody. BF: bright field. Bar: 10 μm.

**
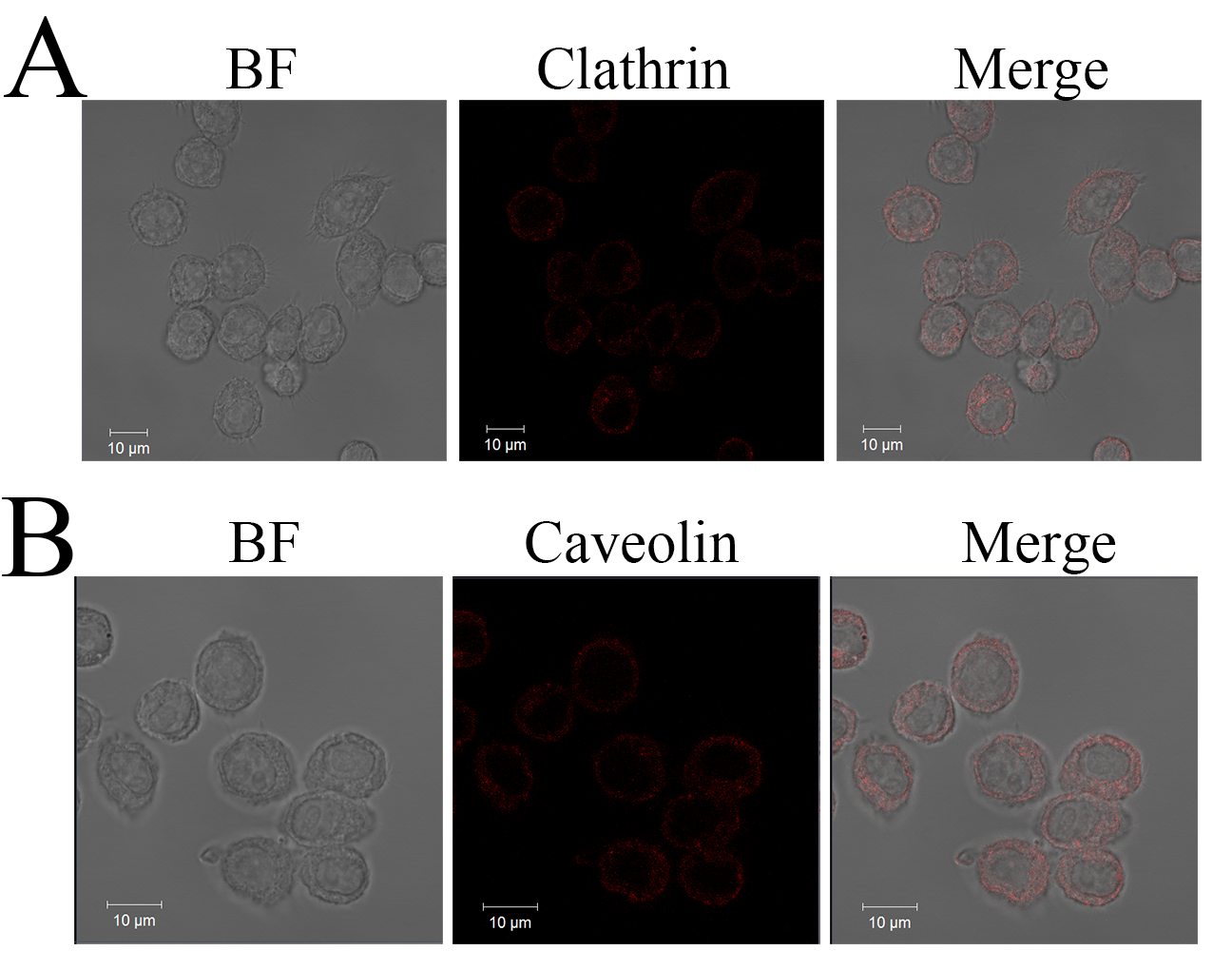
**

**Figure S5.** Co-localization of dead *Edwardsiella tarda* with early and late endosome markers. RAW264.7 cells incubated with dead *E. tarda* TX1G were treated with Alexa Fluor 594-labeled antibody detecting Rab5 (A), Lamp1 (B), or cathepsin D (C). The cells were stained with DAPI and observed with a confocal microscope. White arrows indicate co-localization of bacteria and the respective markers.

**
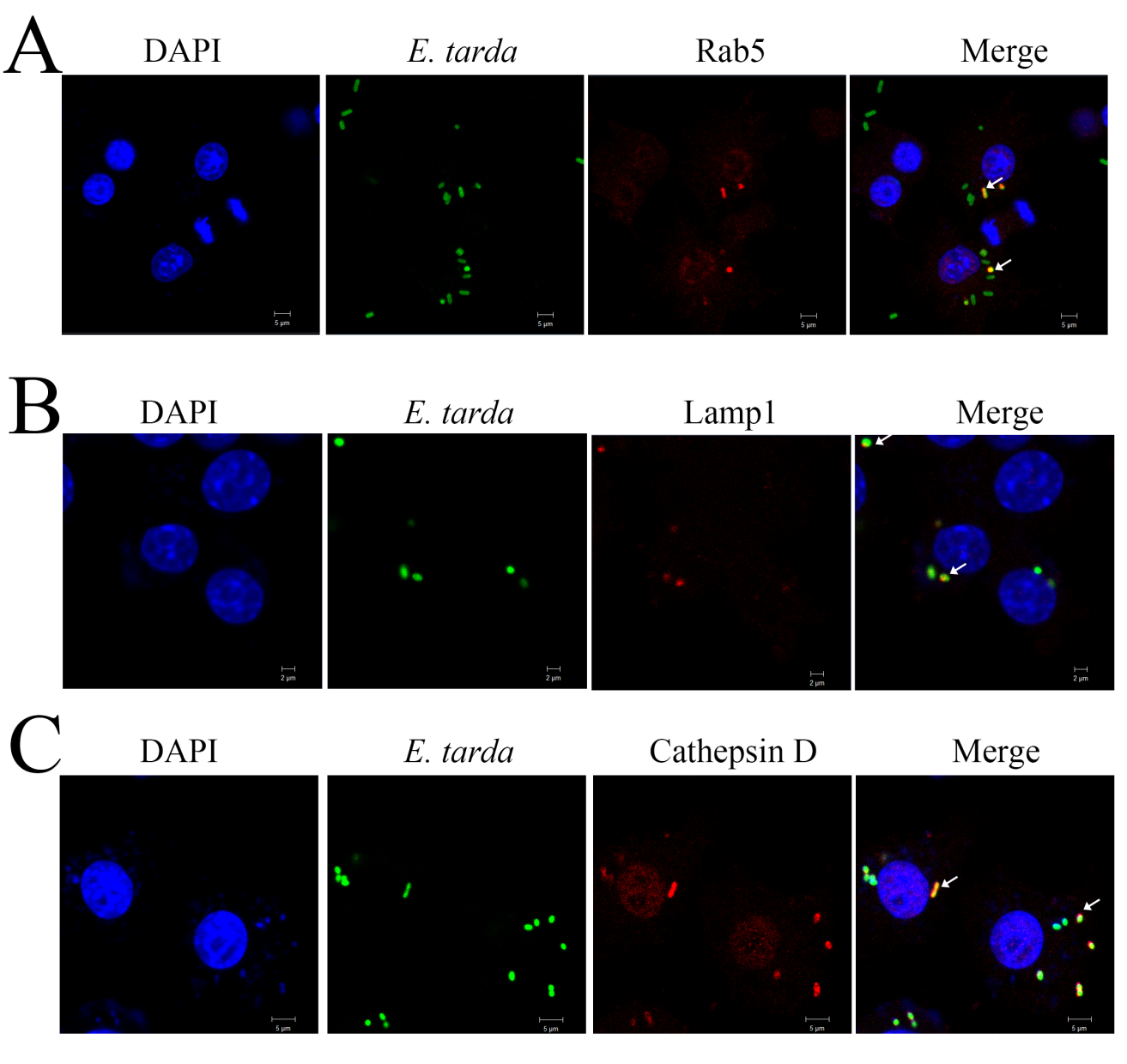
**

**Table S1. siRNAs and primers used in this study**

| SiRNA/Primer | Sequence (5’–3’) | Reference |
| --- | --- | --- |
| Clathrin siRNA | GGCUUCUAAAUAUCACGAAtt | [Zhu et al., 2011](#_ENREF_1)  [Zhu et al., 2011](#_ENREF_1) |
| Caveolin siRNA | CCAUCUACGUCCAUACCUUtt |  |
| Clathrin-RT-F | CTCCTCATCCTCACTGCCATTA | This study |
| Clathrin-RT-R | CAGCGTTCAGCAAACTCATA |  |
| Caveolin-RT-F | GCATCCCAATGGCACTCA | This study |
| Caveolin-RT-R | TGGATCGCAGAAGGTATGG |  |
